# S-1–Based Chemoradiotherapy Followed by Consolidation Chemotherapy With S-1 in Elderly Patients With Esophageal Squamous Cell Carcinoma: A Multicenter Phase II Trial

**DOI:** 10.3389/fonc.2020.01499

**Published:** 2020-08-28

**Authors:** Xin Wang, Xiaolin Ge, Xiaomin Wang, Wencheng Zhang, Haiwen Zhou, Yu Lin, Shuai Qie, Miaomiao Hu, Wei Wang, Ke Liu, Qingsong Pang, Minghe Li, Junqiang Chen, Miaoling Liu, Kaixian Zhang, Ling Li, Yonggang Shi, Wei Deng, Chen Li, Wenjie Ni, Xiao Chang, Weiming Han, Lei Deng, Wenqing Wang, Jun Liang, Nan Bi, Tao Zhang, Wenyang Liu, Jianyang Wang, Yirui Zhai, Qinfu Feng, Dongfu Chen, Zongmei Zhou, Yidian Zhao, Xinchen Sun, Zefen Xiao

**Affiliations:** ^1^Department of Radiation Oncology, National Cancer Center/National Clinical Research Center for Cancer/Cancer Hospital, Chinese Academy of Medical Sciences and Peking Union Medical College, Beijing, China; ^2^Department of Radiation Oncology, Jiangsu Province Hospital (The First Affiliated Hospital With Nanjing Medical University), Nanjing, China; ^3^Department of Radiation Oncology, Anyang Cancer Hospital, Anyang, China; ^4^Department of Radiation Oncology, Tianjin Medical University Cancer Institute and Hospital, National Clinical Research Center for Cancer, Tianjin, China; ^5^Department of Radiation Oncology, Fujian Cancer Hospital, Fujian Medical University Cancer Hospital, Fuzhou, China; ^6^Department of Radiation Oncology, Affiliated Hospital of Hebei University, Baoding, China; ^7^Department of Oncology, Tengzhou Central People's Hospital, Tengzhou, China; ^8^Department of Radiation Oncology, The First Affiliated Hospital of Zhengzhou University, Zhengzhou, China; ^9^Key Laboratory of Carcinogenesis and Translational Research (Ministry of Education/Beijing), Department of Radiation Oncology, Peking University Cancer Hospital & Institute, Beijing, China

**Keywords:** esophageal neoplasms, chemoradiotherapy, radiotherapy, intensity-modulated, chemotherapy, adjuvant, aged, geriatric assessment

## Abstract

**Introduction:** Intensive treatments can often not be administered to elderly patients with esophageal squamous cell carcinoma (ESCC), leading to a poorer prognosis. This multi-center phase II trial aimed to determine the toxicity profile and efficiency of S-1–based simultaneous integrated boost radiotherapy (SIB-RT) followed by consolidation chemotherapy with S-1 in elderly ESCC patients and to evaluate the usefulness of comprehensive geriatric assessment (CGA).

**Patients and Methods:** We prospectively enrolled 46 elderly patients (age ≥ 70 years) with histopathologically proven ESCC. The patients underwent pretreatment CGA followed by SIB-RT (dose, 59.92 Gy/50.4 Gy) in 28 daily fractions administered using intensity-modulated radiotherapy or volumetric-modulated arc therapy. S-1 was orally administered (40–60 mg/m^2^) concurrently with radiotherapy and 4–8 weeks later, for up to four 3-week cycles at the same dose.

**Results:** The median survival time was 22.6 months. The 1- and 2-year overall survival rates were 80.4 and 47.8%, respectively. The overall response rate was 78.3% (36/46). The incidence of grade 3–4 toxicities was 28% (13/46). The most common grade 3–4 toxicities were radiation esophagitis (5/46, 10.9%), nausea (4/46, 8.7%), anorexia (3/46, 6.5%), and radiation pneumonitis (3/46, 6.5%). There were no grade 5 toxicities. CGA identified that 48.8% of patients were at risk for depression and 65.5% had malnutrition.

**Conclusion:** Concurrent S-1 treatment with SIB-RT followed by 4 cycles of S-1 monotherapy yielded satisfactory tumor response rates and manageable toxicities in selected elderly patients with ESCC. Pretreatment CGA uncovered numerous health problems and allowed the provision of appropriate supportive care.

**Clinical Trial Registration:** www.ClinicalTrials.gov, identifier: NCT02979691.

## Introduction

In 2015, China accounted for almost half of all new cases of esophageal cancer in the world (1). The most common type of esophageal cancer in China is esophageal squamous cell carcinoma (ESCC), which accounts for 89% of all esophageal cancer cases (2). As the Chinese population is aging quite rapidly (3), there has been a steady increase in the incidence of ESCC among elderly patients, who account for 30–40% of all ESCC cases (4). The 5-years survival rate of Chinese ESCC patients is 20–30% overall, but this decreases to 15% among elderly patients (5). A significant number of elderly patients with ESCC prefer non-surgical treatments owing to concerns about postoperative morbidity and surgery-related reduction in quality of life. Additionally, many physicians are unlikely to administer aggressive treatment to elderly patients in order to avoid causing severe toxicity reactions. Thus, definitive radiotherapy alone is the most common treatment offered to elderly Chinese patients with ESCC.

We designed this study due to the following reasons: First, we previously conducted a prospective phase I/II trial investigating the safety and feasibility of simultaneous integrated boost radiotherapy (SIB-RT) with concurrent chemotherapy in patients with unresectable ESCC, and determined that the recommended SIB-RT dose was 59.92 Gy/50.40 Gy in 28 fractions (6). Second, the fourth-generation oral fluoropyrimidine S-1 yielded a higher tumor response than and was non-inferior to infusional 5-fluorouracil and UFT (a combination of tegafur and uracil) in phase III trials of patients with gastrointestinal cancer (7, 8). We verified the promising efficacy and safety of S-1 combined with radiotherapy in an adjuvant setting in patients with gastric cancer (9). Third, no consensus has been established regarding target volume delineation in elderly patients with ESCC, and a strict delineation protocol was needed to decrease inter-observer variability in our multicenter phase III ESCC trial (10). Finally, comprehensive geriatric assessment (CGA) has been proposed as a tool for selecting elderly patients for cancer screening and treatment (11, 12). However, this assessment has not been used prospectively in clinical oncology practice in elderly patients with ESCC. Considering the above points, we initiated this multi-center phase II trial to determine the toxicity profile and efficiency of S-1–based SIB-RT followed by consolidation chemotherapy with S-1 in elderly (age ≥ 70 years) ESCC patients and to evaluate the feasibility and usefulness of CGA.

## Materials and Methods

### Eligibility and Ethics

Patients were eligible for inclusion in this prospective multi-center phase II trial if they met the following criteria: [1] age ≥ 70 years, [2] histopathologically proven ESCC, [3] clinical stage II–III or clinical stage IV with metastatic lymph nodes (LNs) in the supraclavicular/celiac trunk area, according to the 6th edition of the American Joint Committee on Cancer classification (13), [4] Karnofsky performance score ≥ 70, [5] Charlson comorbidity index (CCI) score ≤ 3, and [6] normal hematopoietic, hepatic, and renal function.

All patients underwent regular assessments, consisting of physical examinations and complete blood counts performed every week, and liver- and renal-function tests performed every 2 weeks. Follow-up assessments included clinical examination, biochemical tests, barium esophagography, chest/abdominal computed tomography (CT), and cervical ultrasound/CT. Patients were followed up once every 3 months for the first 2 years and every 6 months thereafter. Treatment toxicities were evaluated according to the Common Terminology Criteria for Adverse Events (version 4.0).

We had initially planned to enroll both patients with ESCC and patients with adenocarcinoma of the esophagus. However, despite a long accrual period, we did not meet the sample size for patients with adenocarcinoma. We, therefore, decided to enroll ESCC patients only in this phase II study as well as in our next phase III study. This study was approved by the ethics committee of our hospital and was registered at www.clinicaltrials.gov (NCT02979691). All patients signed informed consent forms.

### Pre-treatment Procedures

The enrolled patients were recommended to undergo nutritional interventions, including feeding tube or gastrostomy feedings before treatment if assessment of their nutritional status indicated a risk of malnutrition. The baseline imaging included endoscopy with biopsy, endoscopic ultrasound, barium esophagography, chest/abdominal CT, cervical ultrasound/CT, and/or positron-emission tomography (PET)/CT. CGA was conducted within a 1-week period between the CT simulation and the first definitive chemoradiotherapy (dCRT) session. The CGA included questions regarding functional status (14, 15), comorbidity (16), cognitive function (17), psychological state (18), nutritional status (19, 20), and social support (Table 1) (21).

**Table 1:** Domains and measures evaluated in the CGA before treatment.

**Domain with measure**	**Item**	***n***	**%**	**Median**	**Range**
**Functional status**					
KPS (0–100)	90	30	65.2		
	80	13	28.3		
	70	3	6.5		
ADL (Barthel index; 0–100)^*^	Independent: score 100	24	58.5	100	50–100
	Minimally dependent: score 75–95	13	31.7		
	Partially dependent: score 50–70	4	9.8		
IADL (Lawton's; 0–5/8)^*^	Independent: score 8 (female) or 5 (male)	25	61.0	7 (female)	5–8 (female)
	Dependent: score < 8 (female) or 5 (male)	16	39.0	5 (male)	2–5 (male)
**Comorbidity**					
CCI (0–37)	0	33	71.7		
	1	12	26.1		
	2	1	2.2		
**Cognitive status**					
MMSE (0–30)^*^	Normal cognition: score 24–30	31	75.6	28	21–30
	Mild cognitive decline: score 18–23	10	24.4		
**Psychological status**					
GDS-5 (0–5)^*^	Not at risk for depression: score 0	21	51.2	0	0–5
	Mild depression: score 1–2	9	22.0		
	Severe depression: score 3–5	11	26.8		
**Nutritional status**					
BMI (kg/m^2^)	Underweight < 18.5	5	10.9	23.1	17–37
	Normal weight: 18.5–23.9	29	63.0		
	Overweight: 24–29.9	10	21.8		
	Obesity: ≥ 30	2	4.3		
MNA-SF (0–14)^*^	Normal nutritional status: score 12–14	12	29.3	10	6–14
	Risk of malnutrition: ≤ 11	29	70.7		
MNA (0-−0)^†^	Normal nutritional status: score 24–30	10	34.5	23	11–28
	Risk of malnutrition: 17–23.5	16	55.2		
	Malnutrition: < 17	3	10.3		
**Social support**					
MOS-SSS (20–100)^*^	NA			84	53–100

* *Calculated using the number of patients with completed questionnaires (n = 41)*.

† *Calculated using the number of patients with a positive MNA screening test (n = 29)*.

*Abbreviations: ADL, activities of daily life; BMI, body-mass index; CCI, Charlson comorbidity index; CGA, comprehensive geriatric assessment; GDS-5, 5-item geriatric depression scale; IADL, instrumental activities of daily living; KPS, Karnofsky performance status; MMSE, Mini Mental State Examination; MNA, Mini Nutritional Assessment; MNA-SF, MNA-short form; MOS-SSS, Medical Outcomes Study—social support survey*.

### Radiotherapy

For the SIB-RT, the planning gross tumor volume (PGTV) and planning target volume (PTV) were administered 59.92 and 50.4 Gy, respectively, in 28 daily fractions of 2.14 and 1.8 Gy, respectively. The clinical target volume (CTV) depended on the location of the primary tumor and was irradiated using involved-field radiotherapy. The CTV included the gross tumor volume (GTV-T) with a radial margin of 0.6–0.8 cm and a longitudinal margin of 3 cm. LNs diagnosed as metastatic or highly suspicious of metastasis (GTV-N) with a margin of 0.5 cm in three dimensions were also included in the CTV for patients whose highest/lowest metastatic LNs were located within 3 cm beyond the primary tumor. For patients in whom the highest/lowest metastatic LNs were located >3 cm beyond the primary tumor, the upper/lower border of the CTV was 0.5 cm superior/inferior to the furthest LN. The PGTV was created by expanding the GTV-T by 1.0 cm longitudinally and 0.5 cm radially and expanding the GTV-N by a uniform 0.5-cm margin. The CTV with a margin of 0.5 cm in three dimensions formed the PTV. For tumors of the cervical esophagus, the supraclavicular LNs were included in CTV. The typical radiographic contouring for three patients with ESCC in the upper, middle, and lower thirds of the esophagus were published in the protocol of our phase III trial (10).

Dose constraints for organs at risk (OAR) were as follows: both lungs, V20 (volume receiving a dose ≥ 20 Gy) <28% and Dmean (mean dose) <16 Gy; heart, V30 < 40% and V40 < 30%; stomach and bowel, V40 < 40%; liver, V30 < 30%; both kidneys, V20 < 30%; and spinal cord, maximal dose for the planning OAR volume = 45 Gy. Radiation was delivered using intensity-modulated radiation therapy (IMRT) or volumetric-modulated arc therapy (VMAT).

### Quality Assurance of Target Volume Delineation

A digital CT-based CTV contouring protocol and three typical cases of elderly patients with ESCCs in the upper, middle, and lower thirds of the esophagus were sent to all the participating centers by the principal institute (10, 22). Before the start of the treatment, each center was asked to provide two sets of CTV contouring images of the three example cases: one based on the contouring principles routinely used at the center and another based on the CTV contouring protocol provided with the example cases. We then compared the CTV contouring images from the different institutes with each other (23), and requested representatives from all centers to attend a workshop on CTV delineation before the accrual of patients in order to enable the identification and discussion of potential problems.

### Chemotherapy Regimen

S-1 was orally administered twice daily on radiotherapy days (total daily dosage, 40, 50, or 60 mg/m^2^ based on the body surface area). At 4–8 weeks after dCRT, oral S-1 administration was to be repeated at the same daily dosage as that used during dCRT. Four cycles of S-1 chemotherapy were to be conducted, with each 3-week cycle involving S-1 administration on days 1–14.

### Study Design and Statistical Analysis

The Response Evaluation Criteria in Solid Tumors (RECIST 1.1) and criteria for barium esophagography for the evaluation of the immediate response to radiotherapy reported by Wan et al. were used to assess the tumor response to treatment (24, 25). Radiographic complete response (CR) was ascertained using barium esophagography and defined as the disappearance of the primary tumor lesion, ulceration, and erosion, and a reduction in the short axis of any metastatic LNs to <10 mm as displayed on CT scans. Partial response (PR) was defined as the disappearance of most of the primary lesion on barium esophagography without obvious distortion and no ulcer outside the lumen. In addition, CT showed an at least 30% decrease in the sum of the diameters of the metastatic LNs, as compared to the sum of the diameters at the baseline.

The primary endpoint was the overall response rate (ORR), which included the radiographic CR and PR rates, as determined within 3 months after dCRT. The secondary endpoints included toxicities, treatment compliance, overall survival (OS), cancer-specific survival (CSS), progression-free survival (PFS), locoregional recurrence-free survival (LRFS), and distant metastasis-free survival (DMFS).

The ORR was predicted to be improved from 40 to 60% based on the results of radiotherapy alone, with a one-sided α = 0.1 and 90% power. A total of 41 patients were required. Assuming a dropout rate of 10%, 46 patients were needed for the phase II study.

The first site of recurrence was used to analyze failure patterns. Locoregional recurrence was defined as relapse of the primary tumor or regional LNs within the radiation field. The time to each outcome was calculated from the start date of dCRT. Statistical analysis was performed using the SPSS for Windows program, version 21.0 (IBM SPSS Inc., Armonk, NY, USA).

## Results

### Patient Characteristics and Response

**Figure 1:**
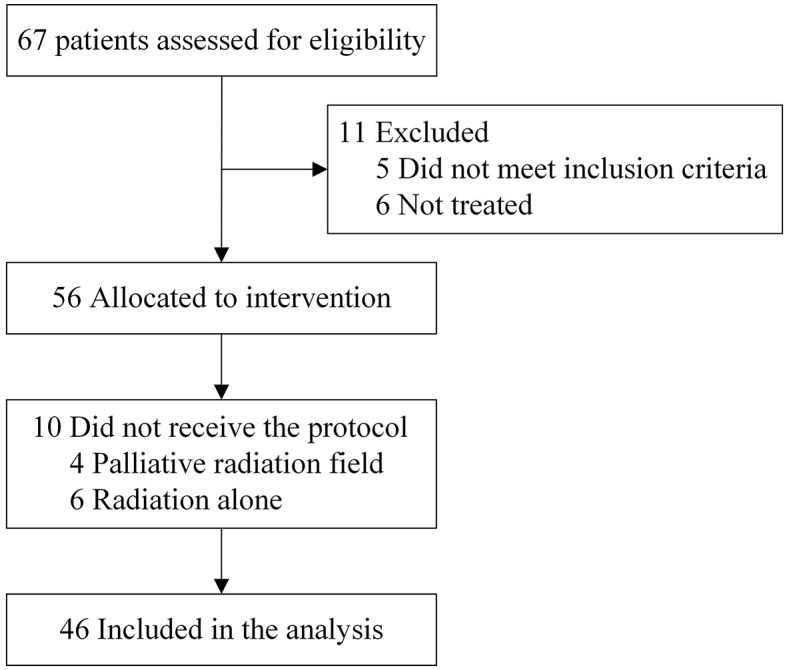
CONSORT diagram.

Between September 2016 and March 2017, 67 patients were screened, and 46 were ultimately recruited from 8 medical centers (Figure 1). The patient characteristics are presented in Table 2. The median age was 75 years (range, 70–91 years), and 54.3% of the patients were diagnosed with clinical stage III/IV ESCC. Before dCRT, 41 (89.1%) patients completed the CGA. The ORR was 78.3% [36/46, 95% confidence interval [CI], 63.6–89.1], including 10 CRs and 26 PRs.

**Table 2:** Patient characteristics.

**Characteristic**	**Total (*n* = 46)**	
	***N***	**%**
Age (years), median (range)	75 (70–91)
Men	33	71.7
Tumor length (cm), median (range)	5 (1.5–10)
**Location of primary tumor (AJCC 6th ed.)**		
Cervical	1	2.2
Upper third	11	23.9
Middle third	24	52.2
Lower third	10	21.7
**T category (AJCC 6th ed.)**		
T1	1	2.2
T2	9	19.5
T3	21	45.7
T4	15	32.6
**N category (AJCC 6th ed.)**		
N0	20	43.5
N1	26	56.5
**M category (AJCC 6th ed.)**		
M0	40	87
M1a	2	4.3
M1b	4	8.7
**Stage (AJCC 6th ed.)**		
IIa	14	30.4
IIb	7	15.2
III	19	41.3
IVa	2	4.3
IVb	4	8.7
Completion of CGA^*^		
Yes	41	89.1
No	5	10.9

* *All 46 patients completed the evaluations for the Karnofsky performance status (KPS), body-mass index (BMI), and Charlson comorbidity index (CCI)*.

*AJCC, American Joint Committee on Cancer; CGA, comprehensive geriatric assessment*.

### Toxicity and Treatment Compliance

The most common toxicities of any grade were as follows: radiation esophagitis (37/46, 80.5%), anorexia (35/46, 76.1%), leukopenia (29/46, 63%), fatigue (29/46, 63%), and nausea (28/46, 60.9%). During dCRT, 13 (28%) of the 46 patients developed grade 3 or 4 toxicities (Table 3). The most common grade 3–4 toxicities were radiation esophagitis (5/46, 10.9%), nausea (4/46, 8.7%), anorexia (3/46, 6.5%), and radiation pneumonitis (3/46, 6.5%). No grade 5 toxicity occurred in this trial.

**Table 3:** Overall toxicities.

**Adverse effects**	**G1-2 (*N*, %)**	**G3 (*N*, %)**	**Total (%)**
Nausea	24 (52.2)	4 (8.7)	60.9
Vomiting	6 (13)	2 (4.3)	17.3
Radiation esophagitis	32 (69.6)	5 (10.9)	80.5
Gastritis	12 (26.1)	0	26.1
Diarrhea	3 (6.5)	0	6.5
Anorexia	32 (69.6)	3 (6.5)	76.1
Fatigue	29 (63)	0	63
Hand-foot syndrome	3 (6.5)	0	6.5
Cough	23 (50)	0	50
Radiation pneumonitis	6 (13)	3 (6.5)	19.5
Hiccup	10 (21.7)	1 (2.2)	23.9
Weight loss	13 (28.3)	1 (2.2)	30.5
Leukopenia	29 (63)	0	63
Neutropenia	16 (34.8)	1 (2.2)	37
Anemia	21 (45.7)	1 (2.2)	47.9
Thrombocytopenia	9 (19.6)	0	19.6
Hypoproteinemia	7 (15.2)	0	15.2
ALT/AST	2 (4.3)	0	4.3

*ALT, alanine aminotransferase; AST, aspartate aminotransferase*.

Seven patients (15.2%) did not complete dCRT because of the following reasons: radiation pneumonitis (grade 2, one patient; grade 3, two patients), vomiting (grade 3, one patient), radiation esophagitis with fever (grade 2, one patient), large radiation field exceeding constraints for OAR (one patient), and refusal of treatment (one patient). Another five patients did not complete the entire course of concurrent S-1 chemotherapy because of grade 3 radiation esophagitis (two patients), grade 3 neutropenia (one patient), grade 2 thrombocytopenia (one patient), and refusal of treatment (one patient).

Consolidation monotherapy with S-1 was continued for at least two cycles in 37 patients (80.4%), at least three cycles in 32 patients (70%), and four cycles in 30 patients (65.2%). The reasons for withdrawal of treatment included refusal to continue the treatment because of adverse events (seven patients), economic reasons (two patients), and sudden death from heart attack (one patient). Six patients did not undergo S-1 treatment after dCRT for the following reasons: refusal of treatment (five patients) and poor recovery from dCRT (one patient).

### Survival Outcomes

Over a median follow-up period of 32.2 months (range, 4.5–39.2 months), the median survival time (MST) was 22.6 months. The 1- and 2-years survival data (in that order) were as follows: OS, 80.4% (95% CI, 74.6–86.2) and 47.8% (95% CI, 40.4–55.2); CSS, 82.3% (95% CI, 76.6–88.0) and 57% (95% CI, 49.5–64.5); PFS, 71.3% (95% CI, 64.6–78.0) and 50.7% (95% CI, 43.2–58.2); LRFS, 79.8% (95% CI, 73.8–85.8) and 67.4% (95% CI, 60.2–74.6); and DMFS, 88.6% (95% CI, 83.8–93.4) and 76.9% (95% CI, 70.0–83.8).

**Figure 2:**
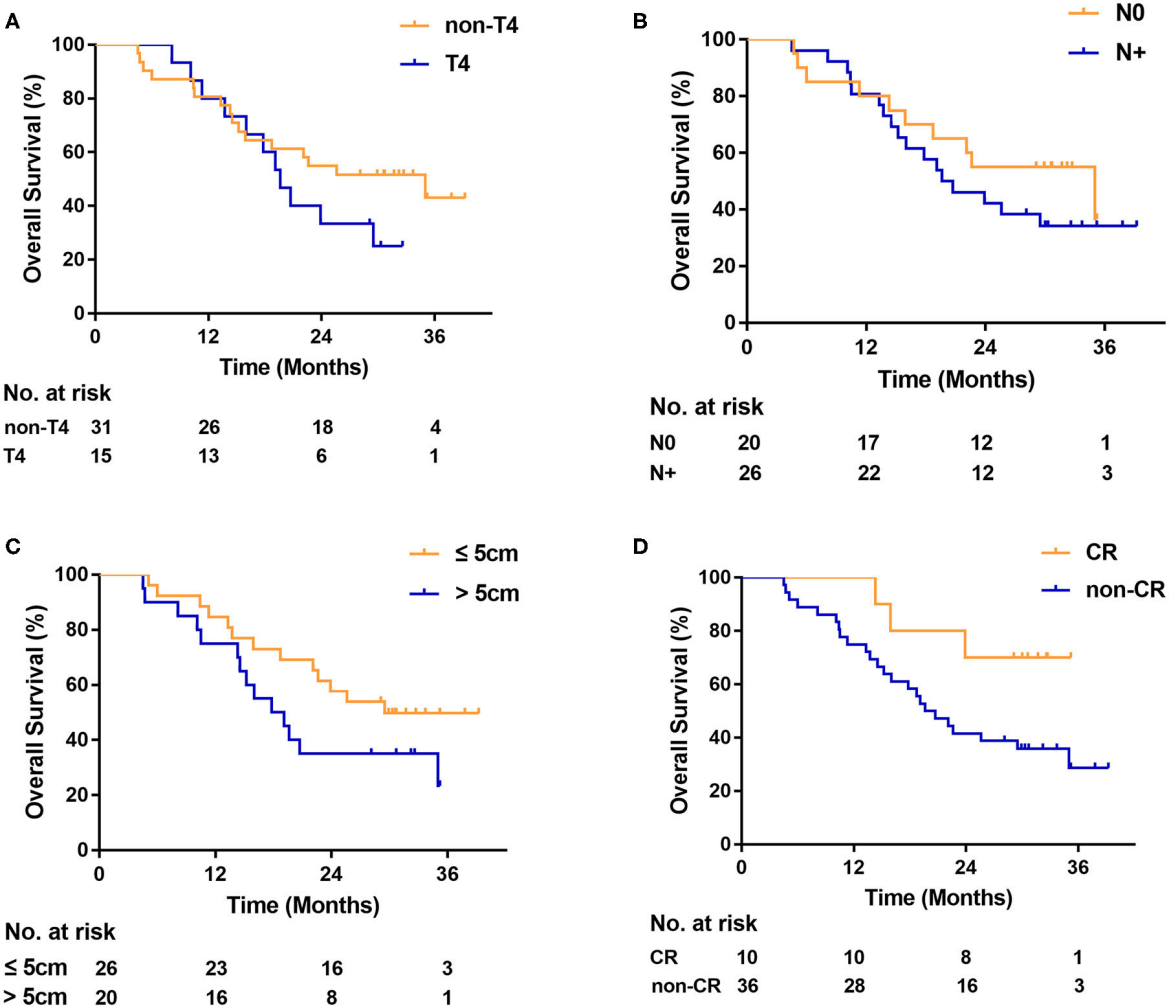
Kaplan-Meier curve of overall survival **(A)** by T category, **(B)** by lymph node status, **(C)** by tumor length, and **(D)** by tumor response. CR indicates radiologic complete response. N0, negative lymph node. N+, positive lymph node.

**Figure 3:**
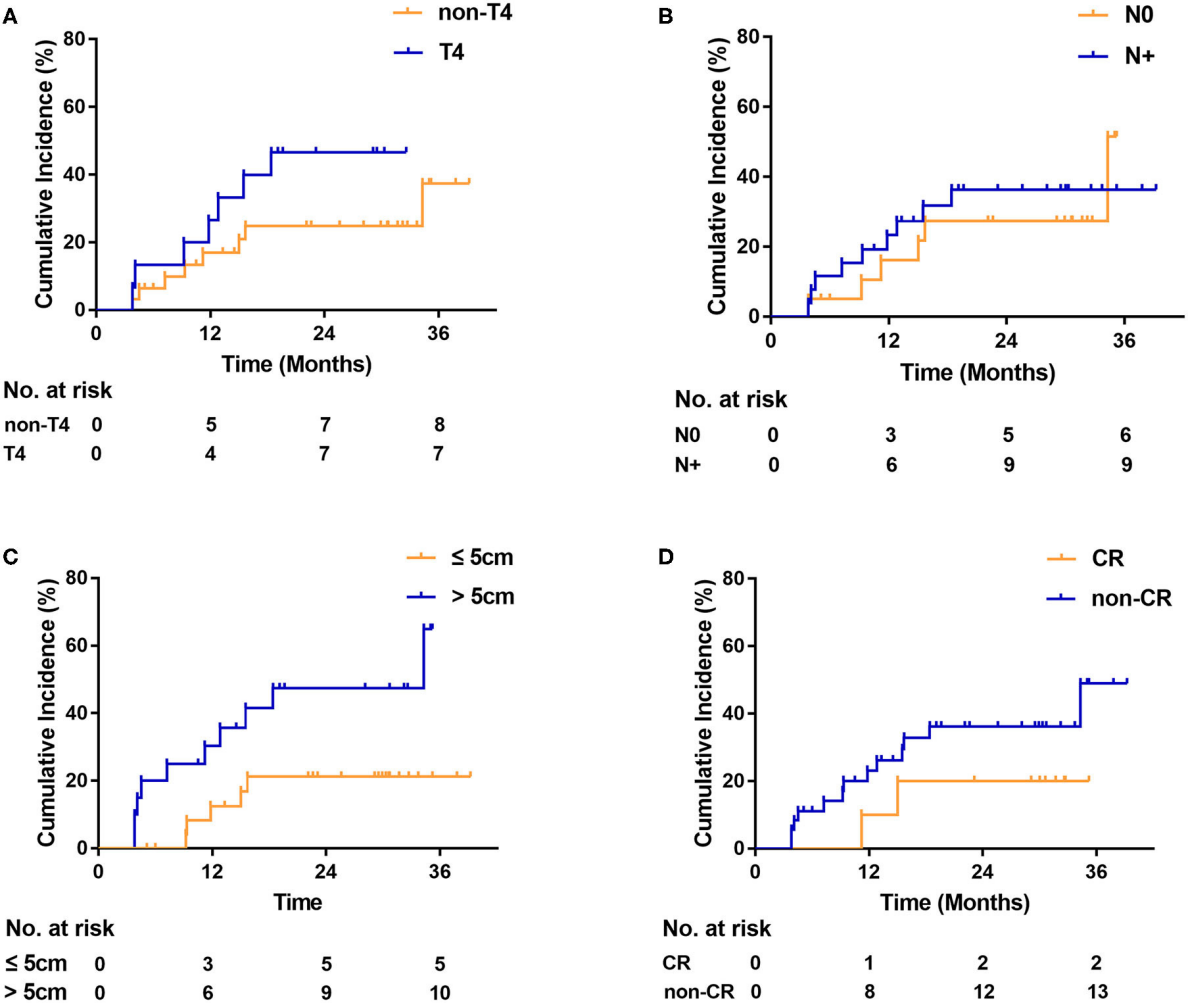
Locoregional recurrence **(A)** by T category, **(B)** by lymph node status, **(C)** by tumor length, and **(D)** by tumor response. CR indicates radiologic complete response. N0, negative lymph node. N+, positive lymph node.

On univariate analysis, OS was not associated with T4 [hazard ratio [HR], 1.6; 95% CI, 0.7–3.7; *P* = 0.22; Figure 2A], LN status (HR, 1.5; 95% CI, 0.7–3.0; *P* = 0.35; Figure 2B), and tumor length (HR, 1.8; 95% CI, 0.8–3.8; *P* = 0.14; Figure 2C). Complete responders had a better OS than non-CR responders, although the difference was not statistically significant (HR, 0.3; 95% CI, 0.1–0.8; *P* = 0.06; Figure 2D). No differences in the locoregional recurrence rate were seen, based on T4 (HR, 2.0; 95% CI, 0.7–6.1; *P* = 0.16; Figure 3A), LN status (HR, 1.2; 95% CI, 0.4–3.4; *P* = 0.70; Figure 3B), and non-CR responders (HR, 2.1; 95% CI, 0.7–7.0; *P* = 0.30; Figure 3D). Patients with tumor length > 5 cm had a significantly higher locoregional recurrence rate than those with tumor length ≤ 5 cm (HR, 3.0; 95% CI, 1.1–8.6; *P* = 0.03; Figure 3C).

No patient had progressive disease at the end of dCRT. Among the 23 patients with progressive disease during the follow-up, 14 patients developed locoregional recurrence, and 8 developed distant metastasis. One patient had both locoregional and distant failure. The most common sites of distant failure were the lungs and intra-abdominal LNs outside the radiation field, which were observed in 4 and 3 patients, respectively. The median time to recurrence was 11.2 months (range, 3.1–34.3 months). The median time from recurrence to death was 3.1 months (range, 0.5–13.7 months). Among the 27 patients who passed away, 23 died of the primary disease, and 4 died of other causes: three due to heart attack and one due to pneumonia.

### Pre-treatment CGA

The results of the CGA are depicted in Table 1. In all, 28.3% of the patients had a CCI score of 1 or 2. We found that 58.5% of the patients were able to independently perform activities of daily living, and 61% could independently carry out instrumental activities of daily living. About one-fourth of the patients (24.4%) experienced mild cognitive decline. The geriatric depression scale (GDS-5) revealed that almost half of the patients (48.8%) were at risk for depression. Nutritional problems were frequent, as two-thirds of the patients (65.5%) were found to have malnutrition according to the Mini Nutritional Assessment.

## Discussion

The results of the present study suggested that SIB-RT at a dose of 59.92 Gy/50.4 Gy with concurrent oral S-1 chemotherapy and followed by S-1 monotherapy had acceptable safety and efficacy in elderly patients with ESCC. The ORR was 78.3%, which met the primary endpoint, and the 2-years OS was 47.8%. This is the first multicenter phase II study to prospectively evaluate the safety and efficacy of this therapeutic regimen with the application of CGA.

Elderly patients have functional limitations in terms of their ability to tolerate intensive treatments such as radical surgery and dCRT with doublet chemotherapy, which are recommended as the standard of care for patients with ESCC (26–28). With the aging of the global, and especially the Chinese, population, it has become increasingly important to find an appropriate therapeutic approach that may be used in elderly patients with ESCC, without causing significant toxicities.

Several studies have assessed the efficacy and safety of doublet chemotherapy-based dCRT in elderly patients with ESCC, but the results were not consistent. Wang et al. found that 54 Gy dCRT with a 3-week regimen of S-1 and cisplatin yielded satisfactory survival outcomes (MST, 18.2 months; 3-years OS, 30.1%), but the treatment-related toxicities (highest grade 3/4, leukopenia, 58%) were relatively high (29). In contrast, Song et al. demonstrated that 60 Gy dCRT with paclitaxel plus cisplatin treatment for selected elderly patients resulted in encouraging survival outcomes (MST, 26.8 months; 2-years OS for stage I–II and III–IV patients, 76.0 and 38.6%, respectively) and tolerable toxicities (highest grade 3/4, leukopenia, 30.5%). In our institutional experience, only 70% of ESCC patients aged ≤ 70 years could complete at least 5 weekly doublet chemotherapy sessions (paclitaxel plus nedaplatin) during dCRT (6).

S-1 has been widely used in East Asia since several studies have reported promising results of S-1–based combination therapy or monotherapy in patients with digestive system tumors (7, 30, 31). Our previous phase I/II study showed that concurrent S-1 chemotherapy (dose, 80 mg/m^2^/d) with SIB-RT after surgery for gastric and esophagogastric junction cancer had a low rate of toxicity (9). Considering these results and the poor tolerance of elderly patients, we used S-1 monotherapy in the present study and reduced the dose to 40–60 mg/m^2^/d based on the body surface area. The survival outcomes in our study were comparable to those in the aforementioned studies (MST, 22.6 months; 2-years OS, 47.8%), and the occurrence rate of severe hematologic toxicities was significantly lower (grade 3/4 leukopenia, 0 patients).

Compared to adenocarcinoma of the esophagus, ESCC tends to recur locoregionally, and shows a better tumor response to radiation therapy (27, 32). As most local failures occur within the irradiated field (33), a higher radiation dose (≥60 Gy) may be necessary to directly improve tumor control and survival rates in patients with ESCC, according to quite a few retrospective studies (34–36). However, this is inconsistent with some other studies from Western countries. The RTOG 9405 trial failed to demonstrate any improvement in OS with dose escalation (64.8 vs. 50.4 Gy); however, this trial was conducted in the era of two-dimensional radiotherapy, which is now considered antiquated (37). The recent, as yet unpublished ARTDECO study from the Netherlands showed that radiation dose escalation up to 61.6 Gy to the primary tumor using the SIB-RT approach did not result in a significant increase in local control over 50.4 Gy in dCRT for esophageal cancer, which is consistent with the RTOG 9405 findings. An improvement in locoregional control after high-dose radiation (3-years LRFS, 63% vs. 53%, *P* = 0.08) was observed with an increase in toxicity and without any improvement in OS. However, ESCC accounted for 61% of all recruited patients in that study, which may have compromised the locoregional control benefit acquired from dose escalation for this pathological type (38). The guidelines of the Chinese Society of Clinical Oncology recommend a total dose of 60–70 Gy administered using new radiation techniques for definitive radiotherapy for ESCC (39).

Newer radiation techniques like IMRT and VMAT are associated with more favorable toxicity profiles and encourage local control, as compared with historical cohorts that received standard-dose dCRT (6, 40, 41). SIB-RT is generally employed to deliver high radiation doses per fraction to the gross tumor and standard fractional doses to areas considered to be at a low risk of disease in patients with various types of cancers (42–44). We previously reported a prospective phase I/II trial investigating the dose escalation of SIB-RT with a weekly infusion of paclitaxel and nedaplatin as dCRT for non-elderly patients with locally advanced ESCC, and determined that the recommended boost dose to the PGTV was 59.92 Gy (equivalent dose in 2 Gy/f, 60.62 Gy) with a standard dose of 50.4 Gy to the PTV (6). With this regimen, the occurrence rate of grade 3/4 side effects was relatively low (leukopenia, 21%; esophagitis, 15%), and the side effects were manageable; moreover, the survival outcomes were promising (1-year OS, 76.9%; LRFS, 78.8%). Thus, we used 59.92 Gy/50.4 Gy as the dose for definitive radiotherapy in the present phase II study and found similar clinical efficacy (1-year OS, 80.4%; LRFS, 79.8%). Our results are also comparable to the outcomes reported by Chen et al., who applied a higher boost dose (63 Gy/50.4 Gy) and found a significant improvement in local control (1-year LRFS, 70%) and prognosis (1-year OS, 78.3%) among non-elderly patients with unresectable locally advanced esophageal cancer (40).

CGA is defined as a multidisciplinary and diagnostic process focusing on identifying comorbidities, functional capabilities, cognition, emotional status, nutritional status, and psychosocial situation; CGA can help guide treatment decisions for elderly patients to achieve the best possible outcomes (45). It has been reported that the application of the CGA in elderly patients with cancers can decrease treatment toxicities and hospitalization duration, and improve the quality of life (12, 46, 47). Our study found that pretreatment screening with the CGA had a major impact on the detection of unknown geriatric problems, and has brought this assessment to the general oncology departments of public hospitals at the national, provincial, and district levels. Before the initiation of this study, the great majority of the treating physicians in the participating centers were unfamiliar with applying the CGA to their patients. As the study proceeded, awareness of the assessment helped the physicians to enroll appropriate candidates, take much better care of patients with severe depression, and perform nutritional interventions upfront for patients at moderate or high risk of malnutrition. Although all physicians were required to obtain the assessment information to their utmost ability, a small number (11%) of patients refused to fill in the questionnaires because they thought they were too complicated to finish. Further research is needed to identify a simpler, more effective alternative that includes the most relevant tools within the CGA for elderly patients with cancer, as this would help decrease treatment toxicity and may even improve prognosis.

The limitations of this study should be considered. First, due to the difficulty of detailed follow-up in China, especially for patients in less developed areas, as well as the poor application of CGA in most Chinese hospitals, this assessment was only conducted in the present study as a screening tool to identify the health problems of the elderly and to estimate the completion rate of these relatively complicated questionnaires. It had no effect on their cancer care, which is a missed opportunity. It is important to note that a potential significant benefit of this assessment is its use before deciding on a cancer care plan to best tailor treatment to the elderly (48). Second, PET-CT is not part of the standard work-up or staging, as this examination is too expensive for most of our patients and is not covered by insurance in our country, which might bring the accuracy of the staging and evaluation of tumor response in this study into question. In addition, assessing tumor response by CT and barium esophagography may have a lower diagnostic accuracy of clinical tumor response, and ORR was not validated by biopsy. However, these two examinations are most commonly used for tumor evaluation in ESCC patients in China, both at the end of dCRT and during long-term follow-up, due to their popular application and low cost.

In summary, concurrent S-1 treatment with SIB-RT followed by 4 cycles of S-1 monotherapy resulted in a satisfactory tumor response and manageable toxicities in selected elderly patients with ESCC. It is worth noting that pretreatment CGA uncovered numerous health problems in our elderly patients and allowed the provision of appropriate supportive care to these patients. The first multicenter randomized phase III study (3JECROG-P01) comparing the present regimen with SIB-RT alone for ESCC in elderly patients is ongoing, and its results are highly awaited (10).

## Data Availability Statement

All datasets presented in this study are included in the article/supplementary material.

## Ethics Statement

The studies involving human participants were reviewed and approved by the institutional review boards of Cancer Institute and Hospital, Chinese Academy of Medical Sciences (reference number NCC2016 YL-06). The patients/participants provided their written informed consent to participate in this study.

## Author Contributions

ZX and XS: conception and design of the study. XinW, WZ, HZ, YL, SQ, MH, WeiW, KL, QP, MLi, JC, MLiu, KZ, LL, YS, LD, WenW, JL, NB, TZ, WL, JW, YZhai, JL, QF, DC, ZZ, and YZhao: study conduct. WD, CL, WN, XC, and WH: data collection. XinW and WH: data analysis. XinW, XG, and XiaW: drafting the manuscript. All authors: revising the manuscript content and final approval of the version to be submitted.

## Conflict of Interest

The authors declare that the research was conducted in the absence of any commercial or financial relationships that could be construed as a potential conflict of interest.

## Abbreviations

CCI: Charlson comorbidity index
CGA: comprehensive geriatric assessment
CI: confidence interval
CR: complete response
CSS: cancer-specific survival
CT: computed tomography
CTV: clinical target volume
dCRT: definitive chemoradiotherapy
DMFS: distant metastasis-free survival
ESCC: esophageal squamous cell carcinoma
GTV-N: nodal gross tumor volume
GTV-T: gross tumor volume
IMRT: intensity-modulated radiation therapy
LN: lymph node
LRFS: locoregional recurrence-free survival
MST: median survival time
OAR: dose constraints for organs at risk
ORR: overall response rate
OS: overall survival
PET: positron-emission tomography
PFS: progression-free survival
PGTV: planning gross tumor volume
PR: partial response
PTV: planning target volume
SIB-RT: simultaneous integrated boost radiotherapy
VMAT: volumetric-modulated arc therapy.
